# Overexpression of *GmPHR1* Promotes Soybean Yield through Global Regulation of Nutrient Acquisition and Root Development

**DOI:** 10.3390/ijms232315274

**Published:** 2022-12-03

**Authors:** Yanjun Li, Wenjing Ma, Kefei Zhang, Xiaoqian Wang, Ran Liu, Yingzhe Tian, Niannian Ma, Qingsong Zhao, Ruineng Xu, Yongjia Zhong, Hong Liao

**Affiliations:** 1 Root Biology Center, Fujian Agriculture and Forestry University, Fuzhou 350002, China; 2 Hebei Laboratory of Crop Genetics and Breeding, National Soybean Improvement Center Shijiazhuang Sub-Center, Huang-Huai-Hai Key Laboratory of Biology and Genetic Improvement of Soybean, Ministry of Agriculture and Rural Affairs, Institute of Cereal and Oil Crops, Hebei Academy of Agricultural and Forestry Sciences, Shijiazhuang 050035, China

**Keywords:** growth promotion, PHR, nodulation, nitrogen, phosphate

## Abstract

MYB-CC transcription factors (TFs) are essential for plant growth and development. Members of the MYB-CC subfamily with long N terminal domains, such as phosphate starvation response 1 (PHR1) or PHR1-like TFs, have well documented functions, while those with short N terminal domains remain less understood. In this study, we identified a nodule specific MYB-CC transcription factor 1 (*GmPHR1*) in soybean that is different from other canonical PHR family genes in that *GmPHR1* harbors a short N terminal ahead of its MYB-CC domain and was highly induced by rhizobium infection. The overexpression of *GmPHR1* dramatically increased the ratio of deformed root hairs, enhanced subsequent soybean nodulation, and promoted soybean growth in pot experiments. The growth promotion effects of *GmPHR1* overexpression were further demonstrated in field trails in which two *GmPHR1-OE* lines yielded 10.78% and 8.19% more than the wild type line. Transcriptome analysis suggested that *GmPHR1* overexpression led to global reprogramming, with 749 genes upregulated and 279 genes downregulated, especially for genes involved in MYB transcription factor activities, root growth, and nutrient acquisition. Taken together, we conclude that *GmPHR1* is a key gene involved in the global regulation of nodulation, root growth, and nutrient acquisition in soybeans, and is thus a promising candidate gene to target for soybean yield enhancement.

## 1. Introduction

The MYB family of transcription factors (TFs) includes a large number of multifunctional TFs acting throughout plant growth and development in a broad spectrum of metabolic processes, as well as responses to biotic and abiotic stresses [1,2,3]. As members of the MYB superfamily, MYB-CC TFs contain typical MYB DNA-binding domains, along with a conserved coiled-coil (CC) domain and variants in the N terminal sequence ahead of the MYB-CC domain that make this group divisible into two subfamilies [4]. Representatives of the MYB-CC family have been identified in rice [4,5], soybean [6,7] and maize [8,9]. However, these previous studies focused predominantly on canonical PHRs with long N terminal sequences ahead of MYB-CC domains, leaving subfamily members lacking long N terminal sequences largely uncharacterized and poorly understood [4,5,7,10].

To date, several MYB-CC TFs with important roles in phosphate starvation responses have been reported in plants [4,6,11]. Among them, phosphate starvation response 1 (PHR1), a long N terminal MYB-CC, has been identified as the phosphate starvation response master regulator in plants [5,12,13]. Others have reported that MYB-CC TF PHR1 also regulates root architecture [14], the expression of phosphate transporters [15], the exudation of organic acids and phosphatases [16,17], and associations with arbuscular mycorrhizal fungi (AMF) [18,19]. In short, PHR1 is involved in a variety of processes that help plants deal with low soil phosphorus availability [20]. This is in accordance with more general observations of the MYB-CC transcription factors that are also involved in responses to abiotic stressors and enhancing crop yields [4,21,22,23,24].

As the most important leguminous crop, soybean contributes substantial amounts of oil and protein to the diets of humans and livestock globally [25,26,27]. As human and livestock populations increase globally, so too do the demands for proteins and oils [28]. Therefore, enhancing soybean yields is important for ensuring food security as demands for foods increase.

Soybean growth and development require the coordinated acquisition and allocation of mineral nutrients acquired predominantly through roots and carbon fixed through photosynthetically active shoot tissues [29]. Nitrogen and phosphorus are not just the two most important mineral nutrients for plant growth, they are also vital for oil and protein synthesis in soybean seeds [30,31]. As a leguminous crop, soybean commonly establishes symbiotic interactions with rhizobial species in which symbiotic nitrogen fixation from atmospheric N_2_ is catalyzed in nodules that can provide up to 68% of the total nitrogen required for growth and development throughout the soybean lifecycle [31,32,33]. Phosphorus, on the other hand, is acquired in the form of phosphate, which is usually fixed by soil organic matter and positive metal ions, and, therefore, is not readily available for uptake by plants in sufficient quantities to ensure optimal growth and productivity [15]. Furthermore, phosphorus is not only essential for soybean plant growth and development, but also for oil synthesis and symbiotic nitrogen fixation through the regulation of nodulation [34,35]. Therefore, phosphorus is particularly important for soybean productivity, as it also influences nitrogen acquisition through symbiotic nitrogen fixation.

In this study, we cloned two non-canonical MYB-CC transcription factors from the short N terminus subfamily of soybean PHR products, each of which was highly expressed in soybean nodules. We functionally characterized *GmPHR1* and revealed novel functions for it in regulation of growth, nodulation, and nutrient acquisition. These results provide insights into the non-canonical PHR subfamily of MYB-CC and how specific members critically influence nutrient efficiency through the regulation of growth, nodulation, and productivity in soybeans.

## 2. Results

### 2.1. GmPHR1 and GmPHR16 Were Highly Expressed in Nodules and Induced by Rhizobium Infection

**Figure 1:**
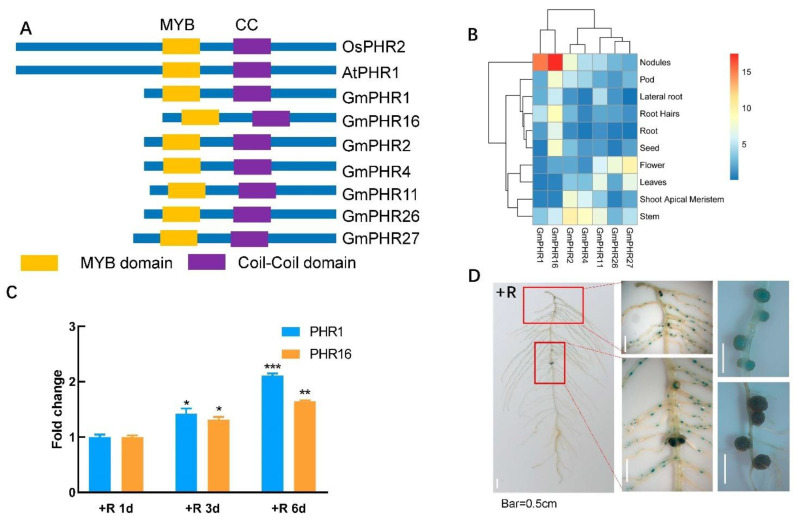
*GmPHR1* and *GmPHR16* were highly expressed in nodules and induced by rhizobium infection. (**A**). Schematic diagram of canonical and non-canonical PHRs with the position of MYB and coiled-coil domains marked in the proteins. (**B**). Expression levels of non-canonical GmPHRs in different tissues of soybean plants were compared using data downloaded from the Phytozome database (https://phytozome-next.jgi.doe.gov/pz/ accessed on 1 September 2021). Expression levels of atypical GmPHRs presented in a heatmap. (**C**). Time course expression analysis of *GmPHR1* and *GmPHR16* inoculated with *Bradyrhizobium elkanii* BXYD3. (**D**). Positive soybean hairy roots carrying *GmPHR1*pro-GUS vector were generated as described in the methods. Hairy roots were inoculated with *Bradyrhizobium elkanii* BXYD3 for one month. Tissue expression patterns were determined with GUS histochemical staining and visualized with stereoscopic microscopes. For (**C**), asterisks represent statistically significantly differences from respective controls in the Student’s *t* test (*: 0.01 < *p* ≤ 0.05, **: 0.001 < *p* ≤ 0.01, ***: *p* ≤ 0.001).

Seven PHR TFs (Transcriptional Factors) with short N terminal amino acid sequences were selected from the MYB-CC family in soybean according to methods outlined in a previous report [7]. Protein amino acid sequence analysis of the selected GmPHRs showed that each of their N terminal sequences ahead of the MYB-CC domain was only a fraction of the length of N terminal sequences from other more typical and better characterized representatives (Figure 1A and Figure S1) [5,7,10]. The expression analysis of seven non-canonical GmPHRs genes on the Phytozome website (https://phytozome-next.jgi.doe.gov/ accessed on 1 September 2021) indicated that *GmPHR1* and *GmPHR16* are the most highly expressed members of the subfamily in nodules (Figure 1B). The nodule specific expression of *GmPHR1* was further inferred using the Soybean eFP Browser (http://bar.utoronto.ca/efpsoybean/cgi-bin/efpWeb.cgi accessed on 15 July 2021) (Supplemental Figure S2A), and then validated in vivo in a qRT-PCR assay (Supplemental Figure S2B). Based upon similar and specific expression patterns, as well as sequence homology and phylogenetic proximity [7], *GmPHR1* and *GmPHR16* were further selected for cloning. Each ORF was amplified with specific primers from cDNA obtained from nodules (Supplemental Figure S3).

Expression analysis in the qRT-PCR assays of whole roots revealed a gradual yet significant induction of expression for both *GmPHR1* and *GmPHR16* subsequent to rhizobium inoculation (Figure 1C). This suggests that these TFs are involved in regulating responses to rhizobium infection. Zooming into subcellular localization in a tobacco leaf transient expression assay pinpointed these two MYB-CC TFs to nuclei (Supplemental Figure S4). To further understand the regulation of these genes, the 2 kb DNA sequence upstream of the initial start codon for *GmPHR1* was cloned as the promoter region and fused with a GUS reporting gene. Histochemical GUS staining of positive transgenic hairy roots produced strong blue signals under low nitrogen and rhizobium inoculation conditions. Meanwhile, relatively lower signals were observed from root tips. In contrast, strong blue signals from nodules suggested that *GmPHR1* was mainly expressed in nodules on soybean roots infected with rhizobium (Figure 1D). Taken together, these results suggest that *GmPHR1* expression varies from expression patterns observed for canonical PHR TFs, and therefore, likely plays specialized roles centered around nodule formation and functionality.

### 2.2. GmPHR1 Overexpression Promotes Soybean Plant Growth and Nodulation

**Figure 2:**
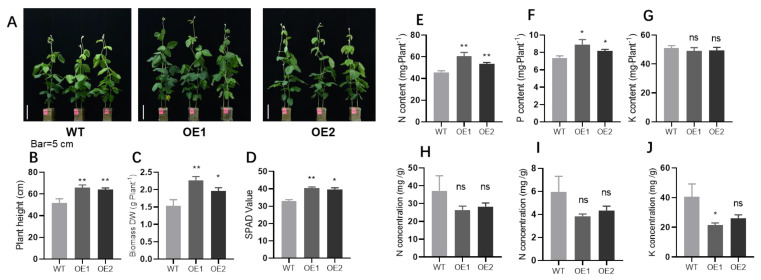
Overexpression of *GmPHR1* promotes soybean growth. (**A**). Growth performance of wild type and transgenic soybean seedling lines overexpressing *GmPHR1* (OE1 and OE2). Bar = 5 cm. (**B**–**D**). Plant height (**B**), biomass (DW: dry weight) (**C**), and leaf SPAD values (**D**) were measured after one-month of growth. *n* = 10. (**E**–**G**). Total nitrogen (**E**), phosphorus (**F**), and potassium (**G**) contents of soybean plants in (**F**). (**H**–**J**). Total nitrogen (**H**), phosphorus (**I**), and potassium (**J**) concentrations of soybean plants in (**F**). For (**B**–**J**), asterisks represent statistically significantly differences from respective controls in the Student’s *t* test (*: 0.01 < *p* ≤ 0.05, **: 0.001 < *p* ≤ 0.01). ns: not significant at 0.05 value.

To illuminate aspects of functionality, two independent transgenic lines of soybean plants overexpressing *GmPHR1* were generated through the introduction of an overexpression vector driven by a 35S promoter (Supplemental Figure S5A,B). In this experiment, *GmPHR1* expression significantly increased in *GmPHR1-OE* lines relative to wild type control plants (Supplemental Figure S5C). In addition, the protein levels of *GmPHR1* were high in *GmPHR1-OE* lines and undetectable in WT plants with anti-Flag antibodies in Western blot testing (Supplemental Figure S5D). The growth performance of *GmPHR1* overexpressing transgenic lines was significantly promoted at the seedling stage, as represented by plant height and dry biomass increases over control plants (Figure 2A–C and Figure S6A–C), suggesting that *GmPHR1* positively regulates soybean plant growth and development. In addition, as *GmPHR1* also harbors conserved MYB-CC domains, it might also share DNA binding properties with canonical PHR TFs. Therefore, we further investigated whether *GmPHR1* influenced nutrient acquisition in soybeans. Here, SPAD values were significantly higher in *GmPHR1-OE* lines than in wild type plants (Figure 2D). Nutrient analysis uncovered increases in nitrogen and phosphorus contents, but not in potassium content, in *GmPHR1-OE* lines over WT plants (Figure 2E–G). Additionally, most of the NPK concentrations in OE lines were lower, but not significantly different, compared to WT plants (Figure 2H–J). In summary, these results suggest that the overexpression of *GmPHR1* may simultaneously enhance the acquisition of nitrogen and phosphate, as well as promote plant growth in soybeans. Moreover, growth promotion in shoots was matched by increases in lateral root density for the basal roots of *GmPHR1-OE* transgenic soybean plants over wild type plants (Supplemental Figure S7). In short, our results suggest that *GmPHR1* may positively regulate soybean plant growth in coordination with the enhancement of nutrient acquisition.

**Figure 3:**
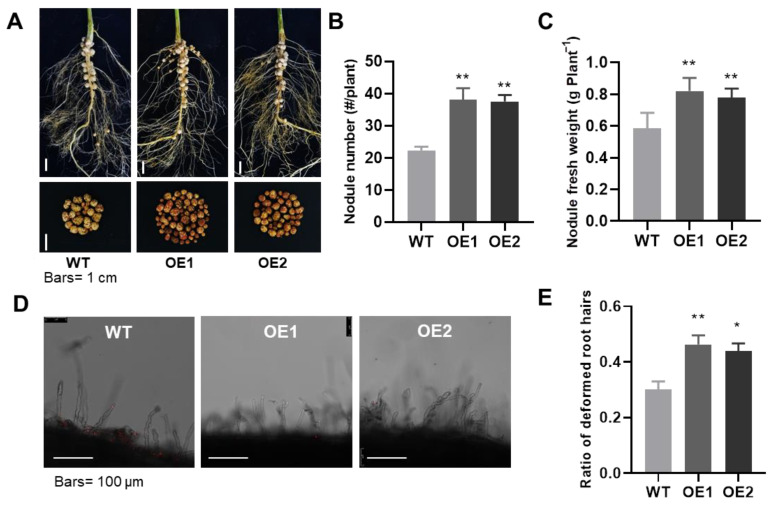
Overexpression of *GmPHR1* promotes nodulation in soybeans. (**A**). Nodules on the roots of wild type and overexpressing transgenic (OE1 and OE2) lines of soybean seedlings. Bar = 1 cm. (**B**,**C**). Nodule number and nodule fresh weight of nodules in (**A**). (**D**). Observed deformed root hairs on wild type and two *GmPHR1-OE* lines inoculated with *Bradyrhizobium elkanii* BXYD3. (**E**). Comparison of deformed root hairs three days after *Bradyrhizobium* inoculation. For (**B**,**C**,**E**), asterisks represent statistically significantly differences from respective controls in the Student’s *t* test (*: 0.01 < *p* ≤ 0.05, **: 0.001 < *p* ≤ 0.01).

Consistent with the observations of whole plant growth and nutrition enhancement, *GmPHR1* was also specifically and highly expressed in nodules (Figure 1B–D). Hence, we presumed that *GmPHR1* might be involved in the regulation of nodulation in soybeans. Results showed that nodule number and nodule fresh weight were significantly higher in the two independent *GmPHR1-OEs* than in the wild type plants (Figure 3A–C), which suggested the positive regulation of nodulation by *GmPHR1* in soybeans. To check whether *GmPHR1* influenced the early aspects of infection in the nodulation process, we observed root hairs and calculated the proportion of deformed hairs for both wild type and *GmPHR1-OE* transgenic lines. In this experiment, the proportion of deformed root hairs was higher in *GmPHR1-OE* lines than in wild type soybeans (Figure 3D,E). This further supported the conclusion that *GmPHR1* promotes nodulation, and further suggested that this regulation occurs, at least in part, during the rhizobium infection phase.

### 2.3. Overexpression of GmPHR1 Increased Soybean Yield under Field Conditions

**Figure 4:**
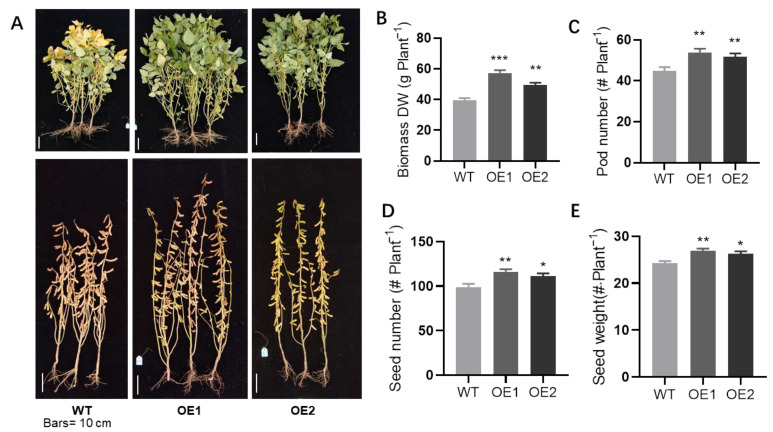
*GmPHR1* overexpression promotes growth and increased yield under field conditions. (**A**). Growth performance of wild type and *GmPHR1-OE* transgenic soybean plants grown under field conditions at the R6 and R7 stages. Bars = 10 cm. (**B**,**C**). The biomass (DW: dry weight) and number of pods per plant for wild type and *GmPHR1-OE* transgenic soybean plants grown under field conditions. (**D**,**E**). The seed number and seed weight per plant of soybean in (**A**). For (**B**–**E**), asterisks represent statistically significantly differences from respective controls in the Student’s *t* test (*: 0.01 < *p* ≤ 0.05, **: 0.001 < *p* ≤ 0.01, ***: *p* ≤ 0.001).

Given that *GmPHR1* positively regulated soybean growth and nodulation, we conducted experiments to check whether *GmPHR1-OEs* could ultimately increase soybean yield. Preliminary pot culture testing showed that plant height and node number were significantly increased in *GmPHR1-OE* lines (Supplemental Figure S8A–C). In the pot culture assay, internode length was significantly increased only in OE1 (Supplemental Figure S9D). Upon maturity, pod number, seed number, and seed weight per plant were all higher in *GmPHR1-OEs* than in wild type soybean plants (Supplemental Figure S9E–G). Continuing tests in field trials revealed that *GmPHR1* overexpressing lines exhibited later maturation phenotypes with taller plants (but showed no significant influence on the flowering time), more nodes, and longer internal node length than wild type control plants (Figure 4A and Figure S9). By the end of maturity, pod number, seed number, and seed weight per plant were all significantly higher in *GmPHR1-OE* lines (Figure 4C–E) than in wild type controls, with no significant influence on the 100 seed weight and an only slightly increased branch number in OE1 but not in OE2 (Supplemental Figure S10). Taken together, these observations strongly indicate that *GmPHR1* may influence growth from early in the lifecycle, and it may continue affecting growth and development through maturity to yield production. In short, *GmPHR1* could be useful for promoting plant growth and high yield in soybeans.

### 2.4. GmPHR1-OE Causes Global Transcriptome Reprogramming in Soybeans

**Figure 5:**
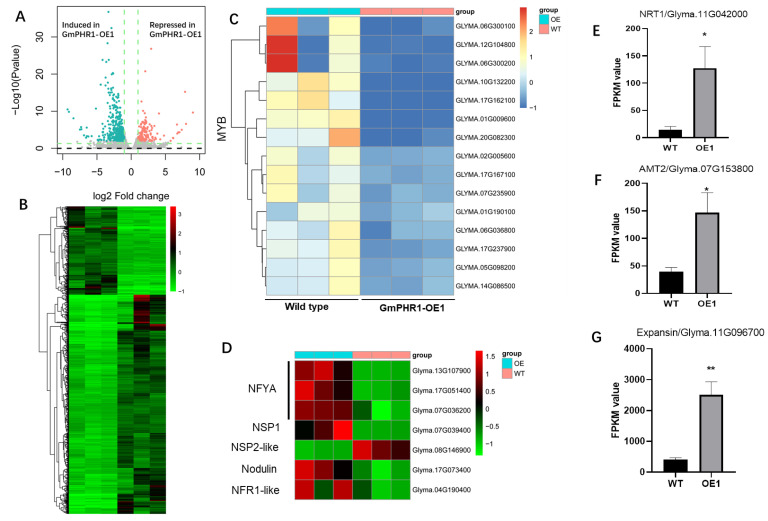
Overexpression of *GmPHR1* induces global transcriptional reprogramming in soybeans. (**A**,**B**). Differentially expressed genes (DEG) compared between wild type and *GmPHR1-OE*1 transgenic lines in a volcano plot (**A**) and heatmap (**B**). Significant expression change threshold was a fold change > 2 and an FDR adjusted *p* value < 0.05. (**C**). MYB transcriptional factors identified among DEGs. (**D**). Significant changes of genes relative to symbiotic signaling genes in soybeans between WT and OE. (**E**–**G**), Comparison of genes acting in nitrogen acquisition (**E**,**F**) and growth regulation (**G**) between wild type and *GmPHR1-OE*1 transgenic plants. For (**E**–**G**), asterisks represent statistically significantly differences from respective controls in the Student’s *t* test (*: 0.01 < *p* ≤ 0.05, **: 0.001 < *p* ≤ 0.01).

To further investigate the mechanisms underlying growth promotion in *GmPHR1-OE* lines, we compared the root transcriptomes of wild type and *GmPHR1-OE* lines in RNA-seq analysis. Here, 1028 genes were significantly influenced by the overexpression of *GmPHR1*, including 749 that were upregulated and 279 that were downregulated (Figure 5A,B). The set of induced genes included a cluster of MYB transcription factors (Figure 5C), suggesting that *GmPHR1* might influence growth through the regulation of plant MYB transcription factors. More importantly, the results from the RNA-seq profile also suggested that the expressions of some genes relative to symbiotic signaling marker genes, such as Nuclear transcription factor Y (NFYA), Nodulation-signaling pathway 1 (NSP1), Early nodulin gene, and Nod factor receptor 1-like (NFR1-like) gene, were all significantly increased in the *GmPHR1-OE* lines [36,37,38,39]. While Nodulation-signaling pathway 2-like (NSP2-like) marker genes were significantly inhibited in *GmPHR1-OE* lines (Figure 5D), other induced genes also appeared to act in nitrogen acquisition (NRT1/*Glyma.11G042000* and AMT2/*Glyma.07G153800*) (Figure 5E,F), and cell growth and expansion (*Glyma.11G096700*) (Figure 5G).

Overall, in this study, we identified a non-canonical MYB-CC transcription factor *GmPHR1* that regulates lateral root growth and nodulation, and in overexpression lines increases lateral root density, enhances biological nitrogen fixation through increases in nodule numbers, and enhances the acquisition of nitrogen and phosphate. All of these responses involve over a thousand differentially expressed genes producing multiple families of regulatory and nutrient acquisition products that ultimately act in coordination to increase soybean yield.

## 3. Discussion

Previously characterized MYB-CC transcription factors have been PHR or PHR-like TFs, which were characterized as central regulators of phosphate starvation responses. In this study, we cloned and characterized a non-canonical MYB-CC *GmPHR1* from the GmPHR-like TF family, according to their amino acid sequences and specific expression patterns (Supplemental Figures S1 and S2). Functional studies suggested that *GmPHR1* might be involved in the regulation of lateral root initiation and nodule formation in roots, as well as influence shoot growth and plant defense genes at the transcriptional level, while phosphate starvation response genes were unaffected, which is distinctive from canonical PHR transcription factors that regulate phosphate starvation response genes [4,5,10,13].

Functional studies were also conducted for *GmPHR1* in two independent transgenic *GmPHR1-OE* lines. Both lines exhibited plant growth promotion capacities in the seedling stage (Figure 2A and Figure S6A) and at maturity (Supplemental Figure S9A,B). These observations were consistent across pot culture and field experiments (Figure 4 and Figure S9). Growth promotion in *GmPHR1-OE* lines was accompanied by increases in plant height and node number (Supplemental Figure S9) or increases in internal node length (Supplemental Figures S6A, S9D and S10C). In roots, *GmPHR1* positively regulated the initiation of lateral roots and lateral root density (Supplemental Figure S7), which is consistent with the expression observed in lateral root primordia and root tips (Supplemental Figure S2A). The regulation of root growth, plant height, and the internal node length might be due to an increase in the expression of signaling related genes, such as auxin-responsive protein (*Glyma.19G258800*) [40,41] and a cell growth regulating expansion gene (*Glyma.11G096700*) [42,43].

The regulation of lateral roots by *GmPHR1* is distinct from regulation by canonical PHR transcription factors, which mainly increase root hair density and root hair length, but not lateral root density [5,44]. In addition, overexpression of *GmPHR1* also increased nodule numbers and nodule fresh weights (Figure 3A–C), which is consistent with the high expression of *GmPHR1* noted in nodules (Supplemental Figure S2), as well as the induction of expression by the symbiotic signaling pathway (Figure 1B). Increases in the proportion of deformed root hairs were observed in *GmPHR1-OE* lines over wild type lines subsequent to rhizobium infection (Figure 3D,E). In addition, genes related to nodulation symbiotic signaling pathway were mostly upregulated in the *GmPHR1-OE* (Figure 5D). These might explain the promotion of nodulation observed in *GmPHR1-OE* lines. Taken together, our results clearly indicate that the overexpression of *GmPHR1* simultaneously promotes both aboveground and belowground growth in soybean.

These results also suggest that *GmPHR1-OEs* increase phosphate and nitrogen acquisition, but not potassium acquisition (Figure 2E–G), which might result in simultaneous increases in both nitrogen and phosphate acquisition efficiency. One potential explanation is that *GmPHR1* overexpression increases nodule number and thus enhances biological nitrogen fixation while simultaneously increasing phosphate acquisition through increases in lateral root density, with cross-talk between nitrogen and phosphate pathways possibly playing a role [45,46]. More interestingly, our RNA-seq results suggest that typical phosphate starvation signaling genes are not among the DEGs observed with the overexpression of *GmPHR1* (Supplemental Table S2). The DEGs that were observed were mostly clustered in families of genes apparently functioning in defense responses, nitrogen acquisition, and growth (Figure 5A,B). This indicates that the observed promotion of phosphate acquisition was not due to the regulation of the phosphate starvation singling pathway, but rather occurred in coordination with the promotion of root growth and nodulation. Taken together, these results suggest that the regulatory functions of *GmPHR1* are distinctive from those of canonical PHR1 products in the regulation of phosphate starvation responses in plants [4,5,10,12].

These results also suggest that soybean yield increases significantly with the overexpression of *GmPHR1*. This might be explained by the contributions of enhanced nitrogen and phosphate acquisition through symbiotic nitrogen fixation, along with increases in root growth (Figure 3 and Figure S7), as well as upregulation of nitrogen acquisition genes (Figure 5E) and increases in the number of shoot nodes and subsequently developed pods (Figure 4C and Figure S9C,D). Meanwhile, the tolerance of *GmPHR1-OE* lines to abiotic and biotic stresses might also be altered, as noted by the upregulation of a number of MYB transcription factors, such as *Glyma.10G132200*, which is a homologue with AtMYB71 in Arabidopsis and involved in abscisic acid responses [47]. *Glyma.17G167100* and *Glyma.06G036800* are homologues with *AtMYB70* and *AtMYB77*, which are reported to be the regulators of root, lateral root growth and development [48,49]. Taken together, the range of complementary and consistent results presented in this study strongly suggest that *GmPHR1* is involved in plant growth regulation that is realized through increased nitrogen and phosphate efficiency, and that it might also regulate tradeoffs between immunity and growth.

## 4. Material and Methods

### 4.1. Plant Materials and Growth

Soybean (*Glycine max* (L.) Merr) cultivar Williams 82 was used for explants for the generation of *GmPHR1-OE* transgenic lines. Williams 82 plants were included in experiments as the control treatment.

For pot cultures, sterile vermiculite and perlite were mixed in a 1:1 ratio and loaded in the pots. Soybean seeds of wild type (Williams 82) and transgenic *GmPHR1-OE* lines were sown with three seeds per pot. A total of nine pots were prepared for each treatment. Seven days after the initiating germination, seedlings were thinned to one plant per pot, with all remaining plants being of similar growth performance. Pots were placed in a growth chamber for 40 days (day/night: 14 h/10 h, 26 °C/24 °C) prior to sampling. During plant growth, pots were supplied with 100 mL of soybean nutrient solutions every 3 days [50].

For outdoor pot cultures, wild type and transgenic *GmPHR1-OEs* soybean plants were grown in pots filled with soils collected from the Yangzhong field experimental station of Fujian Agriculture and Forestry University [51]. Seeds were sown as described above, with a total of nine pots prepared for each treatment. Pots with seedlings were placed under natural light conditions until they reached maturity (May to July, 2019). During plant growth, normal nutrient solution was supplied according to plant needs.

Field trials were also conducted to test for the influence of *GmPHR1* overexpression on the growth and yield of soybean plants. Soybean plants were planted in a spilt plot design with plots arranged in three replicates of randomized complete blocks within the split plots. A total of 30 seeds of wild type soybean seeds and *GmPHR1-OE* transgenic seeds were sown in 3 m plots with a 20 cm planting distance and 40 cm distance between rows. Plants were grown until the R6 stage for sampling plants and measuring grain yield. No fertilizer was applied during soybean growth. Field management, irrigation, and pest control followed local practices.

### 4.2. Vector Construction

The ORF of *GmPHR1* was amplified form nodule cDNA and cloned into a *pEASY-BLUNT* vector (TransGen Biotech, Beijing, China). After sequencing, the ORF of *GmPHR1* was sub-cloned to the *5941-35S-3×Flag* using the AscI restriction site. The resulting overexpressing vectors *pFGC5941-35S-GmPHR1-3×Flag* were generated. To investigate the tissue expression patterns of *GmPHR1*, the DNA region 2 kb upstream of the *GmPHR1* translation initiation site was cloned as the promoter region and fused with the GUS reporting gene in the *pFGC5941-GUS* vector to generate *pFGC5941*-*proGmPHR1-GUS* constructs. The primers used for vector construction are listed in Supplemental Table S1.

### 4.3. Generation of Stable Transgenic Plants

The construct *pFGC5941-35S-GmPHR1-3×Flag* was transformed into A. tumefaciens strain EHA105 as previously described [52]. Soybean cultivar Williams 82 was used as the explant for the generation of transgenic plants as described by Wang et al., 2009 [16]. Transgenic plants were identified with Bar resistance. The *Bar* gene was amplified using *Bar-F* and *Bar-R*, along with *35S-F* and *GmPHR1-test-R* (Supplemental Table S1).

### 4.4. Generation of Hairy Roots and GUS Staining

The construct *pFGC5941-GmPHR1pro-GUS* was transformed into *A. rhizogenes* strain K599 as described in Xu et al., 2021. Then, hairy roots were inoculated with *B. japonicum* strain USDA110 (OD600 = 0.8) [44]. Hairy roots were co-cultured with rhizobium for one month in a growth chamber (14 h of light, 28 °C, and 60% RH). Hairy roots were then harvested for GUS staining as described in Xu et al., 2021 [36].

### 4.5. Western-Blotting

To quantify the expression of *GmPHR1* in overexpression lines, shoots of *GmPHR1-OE* transgenic plants and wild type plants were ground in liquid nitrogen, and total protein was extracted in Pierce IP buffer (Thermo Scientific, Waltham, MA, USA) amended with 1 mM PMSF, 5 mM MG132, and 1× protease inhibitor cocktail (Roche, Basel, Switzerland). Total protein (20 µg) was loaded onto 12% SDS-PAGE gels for electrophoresis and then transferred to NC membranes (Bio-Rad, Hercules, CA, USA) using Trans-Blot Turbo (Bio-Rad). The blotting procedures were conducted according to Zhong et al., 2018 [13]. After washing three times, bound antibodies were visualized with ECL substrate (Millipore, Burlington, MA, USA) using the ChemDoc XRS system (Bio-Rad). The dilution ratio for mouse anti-flag (Sigma-Aldrich, St. Louis, MO, USA) was 1:3000.

To quantify deformed root hairs, transgenic *GmPHR1-OE* soybean seeds were germinated along with wild type soybean seeds in sterile vermiculite, inoculated with 50 mL RFP labeled *Bradyrhizobium* BXYD3, and supplied with nitrogen free nutrient solution. Root hair phenotypes were recorded using a confocal laser scanning microscope (LSM; Carl Zeiss, Oberkochen, Germany) five days after rhizobium inoculation [36]. The ratio of deformed root hairs was calculated as the number of deformed root hairs to the total number of root hairs.

### 4.6. Nitrogen, Phosphorus and Potassium Content Measurements

To measure N, P and K content, plants were dried at 65 °C for two days prior to grinding the dried plant to powder. Then, 0.2 g powder was digested with H_2_SO_4_ to measure total N and P content using a continuous flow analyzer (SAN++). The potassium was measured with Flame Spectrophotometer Sherwood M410 (Sherwood Scientific, Cambridge, UK). The procedures of the operation and computational analysis were performed according to Li et al., 2015 and Wang et al., 2021 [50,51].

### 4.7. Total RNA Extraction and Quantitative Real Time PCR

For time course expression analysis, root samples were collected at 1 day, 3 days, and 6 days after *Bradyrhizobium elkanii* BXYD3 inoculation. Total RNA samples were extracted from soybean roots after treatment with RNAiso Plus reagent (Takara Bio, Kusatsu, Japan) as described in Xu et al., 2021 [36]. Total RNA was treated with DNase to remove genomic DNA. Then, 1 μg of total RNA was used for first strand cDNA synthesis using Oligo dT and random primers and Moloney murine leukemia virus reverse transcriptase according to the manual (TransGen Biotech, Beijing, China). Quantitative real time PCR was performed in 20 μL volumes using methods outlined by Xu et al., 2021 [36]. The soybean GmEF1α gene (Accession no. X56856) was used as the internal reference [50]. Relative expression levels of target genes were calculated using the 2^−ΔΔCT^ method [53]. Primers used for detecting target genes are listed in Supplemental Table S1.

### 4.8. RNA-Seq and Bioinformatics Analysis

Total RNA was extracted as described above. Quality and quantity of RNA was measured using a Nanodrop 2000 spectrophotometer (Thermo Scientific, USA). The sequencing library was constructed using 5 µg of total RNA with a NEB Next^®^ Ultra™ RNA Library Prep Kit (NEB, Herts, UK). The cDNA libraries were quantified using an AMPure XP system (Beckman Coulter, Beverly, MA, USA). Then, sequencing was performed by Novogene Co., Ltd. (Beijing, China).

Raw sequence data were filtered with fastqc software (version: 0.11.9; (http://www.bioinformatics.babraham.ac.uk/projects/fastqc/ accessed on 10 August 2020), and adaptors were removed using Trimmomatic software (version: 0.32) [54]. Sequences were mapped to the genome using STAR software (v2.7.10) [55]. Gene expression quantification was calculated with STAR and RSEM software (v1.3.3) [55,56]. Differences in gene expression were analyzed using DESeq2 [57].

### 4.9. Statistical Analysis

Means and SE values were calculated using GraphPad Prism version 7.0 (GraphPad Software Inc., San Diego, CA, USA; https://www.graphpad.com accessed on 23 June 2018). The two-tailed Student’s *t* test was used to calculate the significance between samples. Duncan’s multiple comparison was used to separate sample means. All statistical analysis was performed using SPSS software version 19 [58], GraphPad Prism (version 7.0), and R (version: 4.0) [59].

## Data Availability

Raw RNA-Seq sequences were deposited in NCBI with the accession number of PRJNA793711 (https://www.ncbi.nlm.nih.gov/bioproject/PRJNA793711).

## Supplementary Materials

The following supporting information can be downloaded at: https://www.mdpi.com/article/10.3390/ijms232315274/s1.
