# Supplementary material for: Overexpression of GmPHR1 Promotes Soybean Yield through Global Regulation of Nutrient Acquisition and Root Development
Source: Int J Mol Sci. 2022 Dec 3;23(23):15274. doi: 10.3390/ijms232315274 (PMC9740814; doi:10.3390/ijms232315274)
Supplement: Supplementary file 1 [file ijms-23-15274-s001.zip › ijms-2021828-supplementary.pptx]

## Slide 1
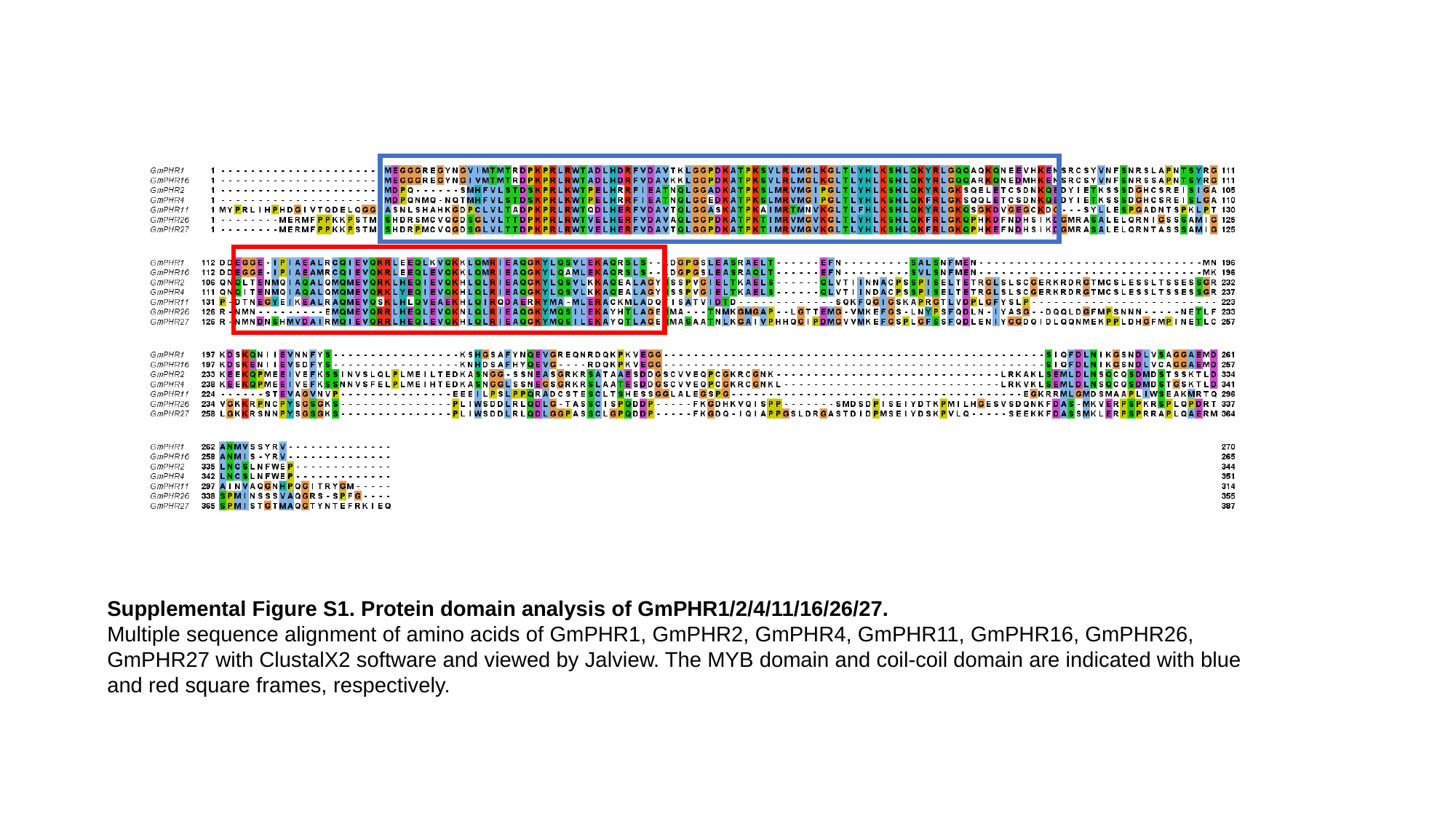

Supplemental Figure S1. Protein domain analysis of GmPHR1/2/4/11/16/26/27.
Multiple sequence alignment of amino acids of GmPHR1, GmPHR2, GmPHR4, GmPHR11, GmPHR16, GmPHR26, GmPHR27 with ClustalX2 software and viewed by Jalview. The MYB domain and coil-coil domain are indicated with blue and red square frames, respectively.

## Slide 2
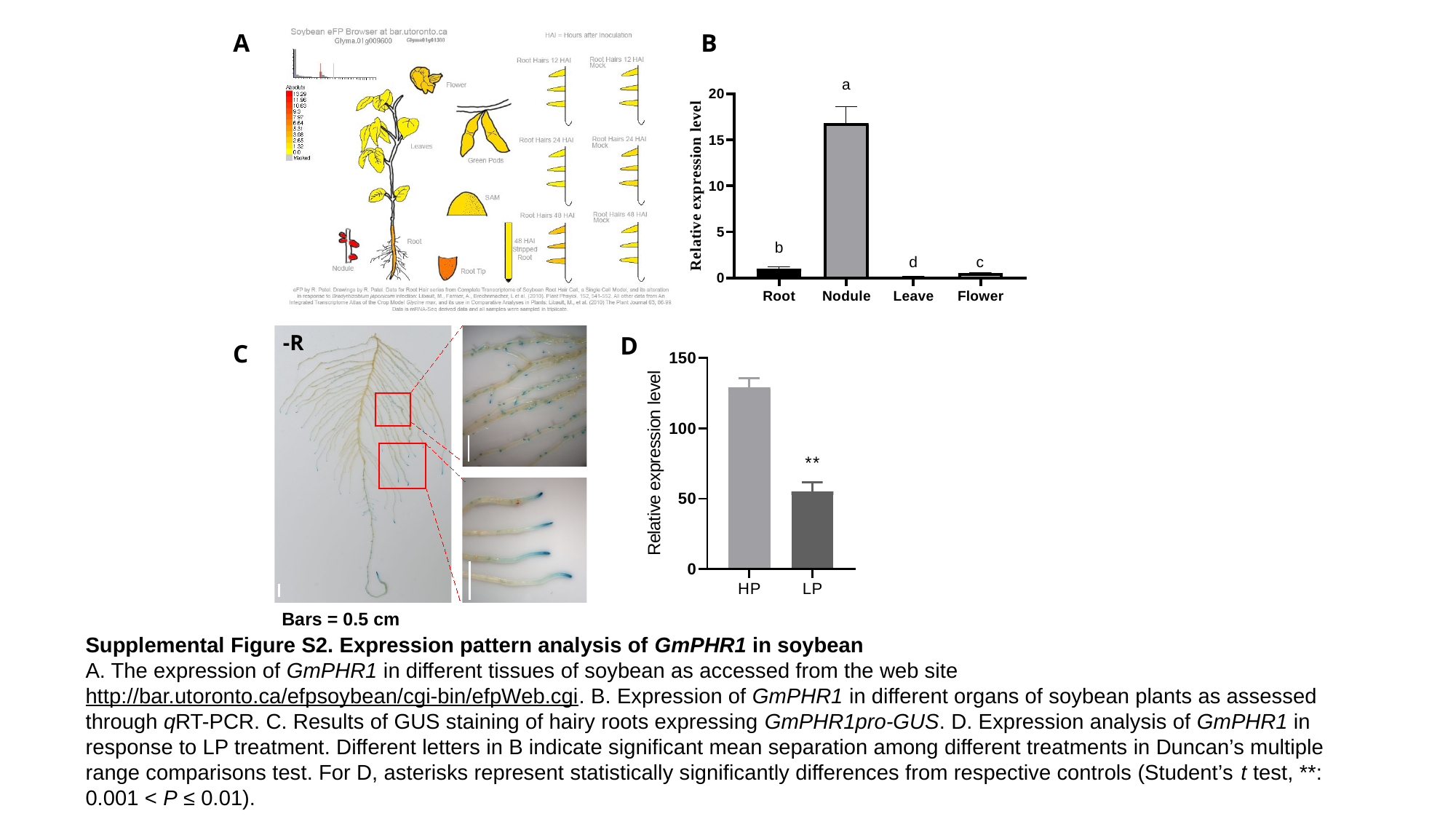

A
B
-R
D
C
Bars = 0.5 cm
Supplemental Figure S2. Expression pattern analysis of GmPHR1 in soybean
A. The expression of GmPHR1 in different tissues of soybean as accessed from the web site http://bar.utoronto.ca/efpsoybean/cgi-bin/efpWeb.cgi. B. Expression of GmPHR1 in different organs of soybean plants as assessed through qRT-PCR. C. Results of GUS staining of hairy roots expressing GmPHR1pro-GUS. D. Expression analysis of GmPHR1 in response to LP treatment. Different letters in B indicate significant mean separation among different treatments in Duncan’s multiple range comparisons test. For D, asterisks represent statistically significantly differences from respective controls (Student’s t test, **: 0.001 < P ≤ 0.01).

## Slide 3
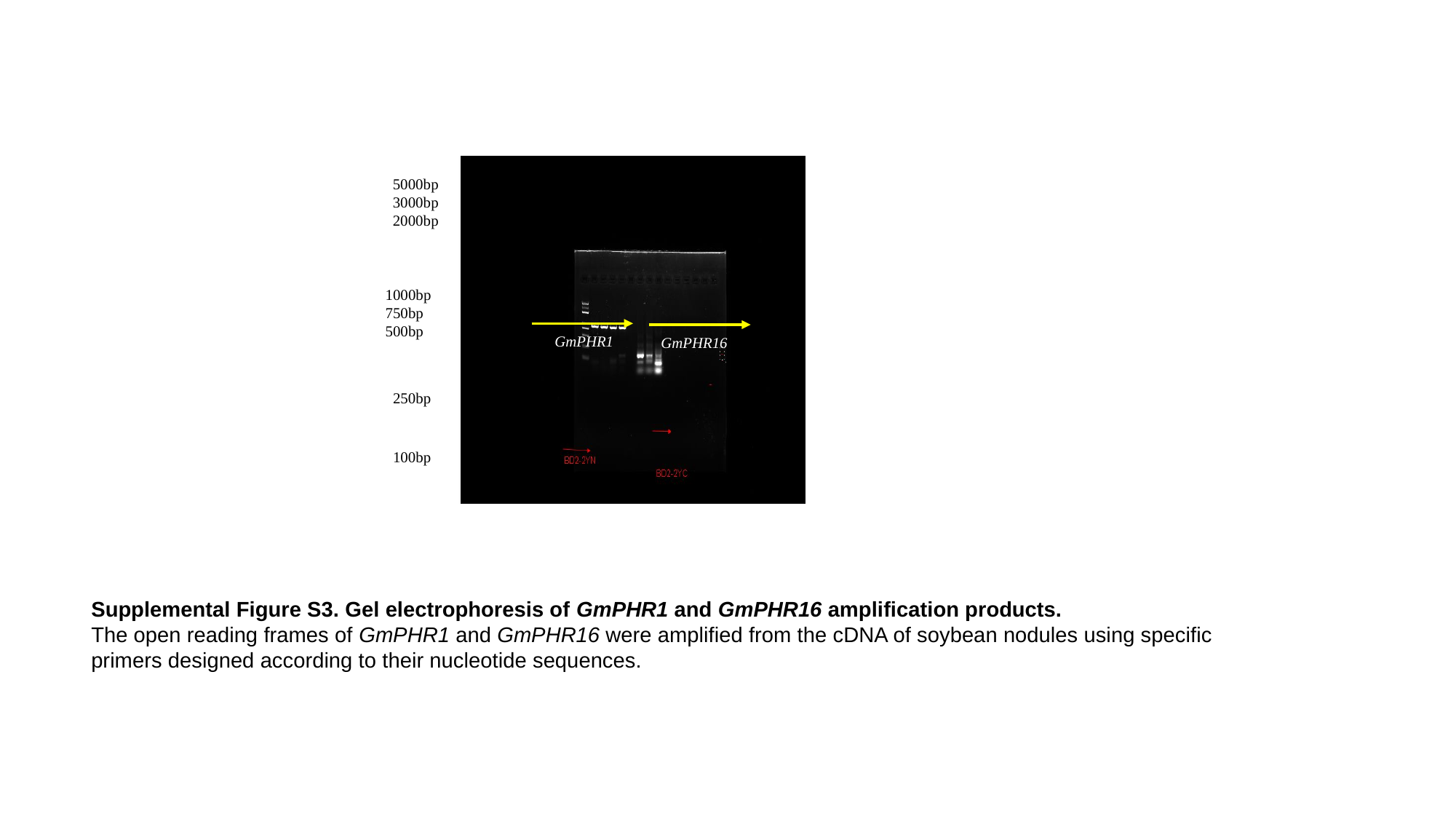

5000bp
3000bp
2000bp
1000bp
750bp
500bp
GmPHR1
GmPHR16
250bp
100bp
Supplemental Figure S3. Gel electrophoresis of GmPHR1 and GmPHR16 amplification products.
The open reading frames of GmPHR1 and GmPHR16 were amplified from the cDNA of soybean nodules using specific primers designed according to their nucleotide sequences.

## Slide 4
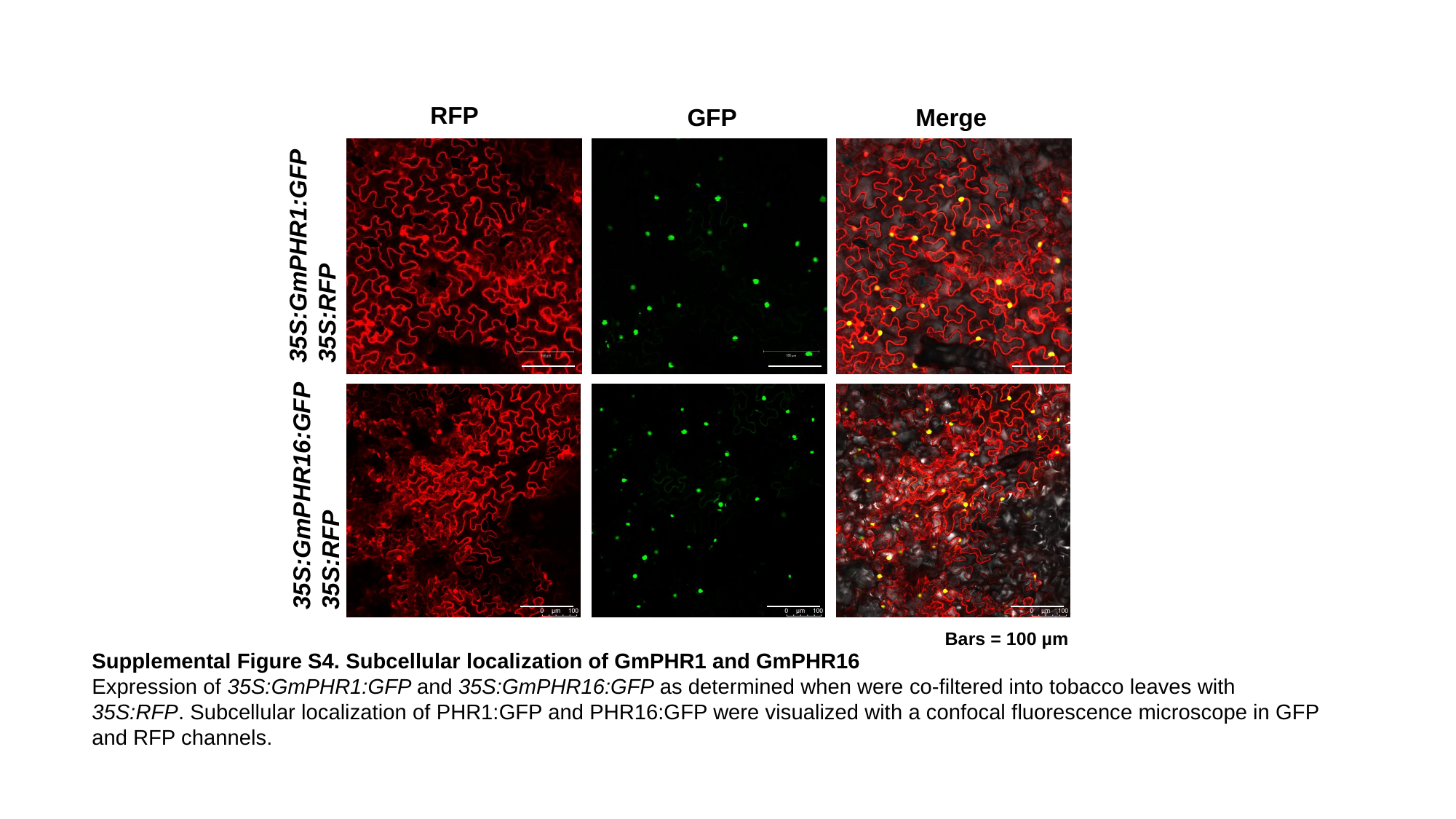

RFP
GFP
Merge
35S:GmPHR1:GFP
35S:RFP
35S:GmPHR16:GFP
35S:RFP
Bars = 100 µm
Supplemental Figure S4. Subcellular localization of GmPHR1 and GmPHR16
Expression of 35S:GmPHR1:GFP and 35S:GmPHR16:GFP as determined when were co-filtered into tobacco leaves with 35S:RFP. Subcellular localization of PHR1:GFP and PHR16:GFP were visualized with a confocal fluorescence microscope in GFP and RFP channels.

## Slide 5
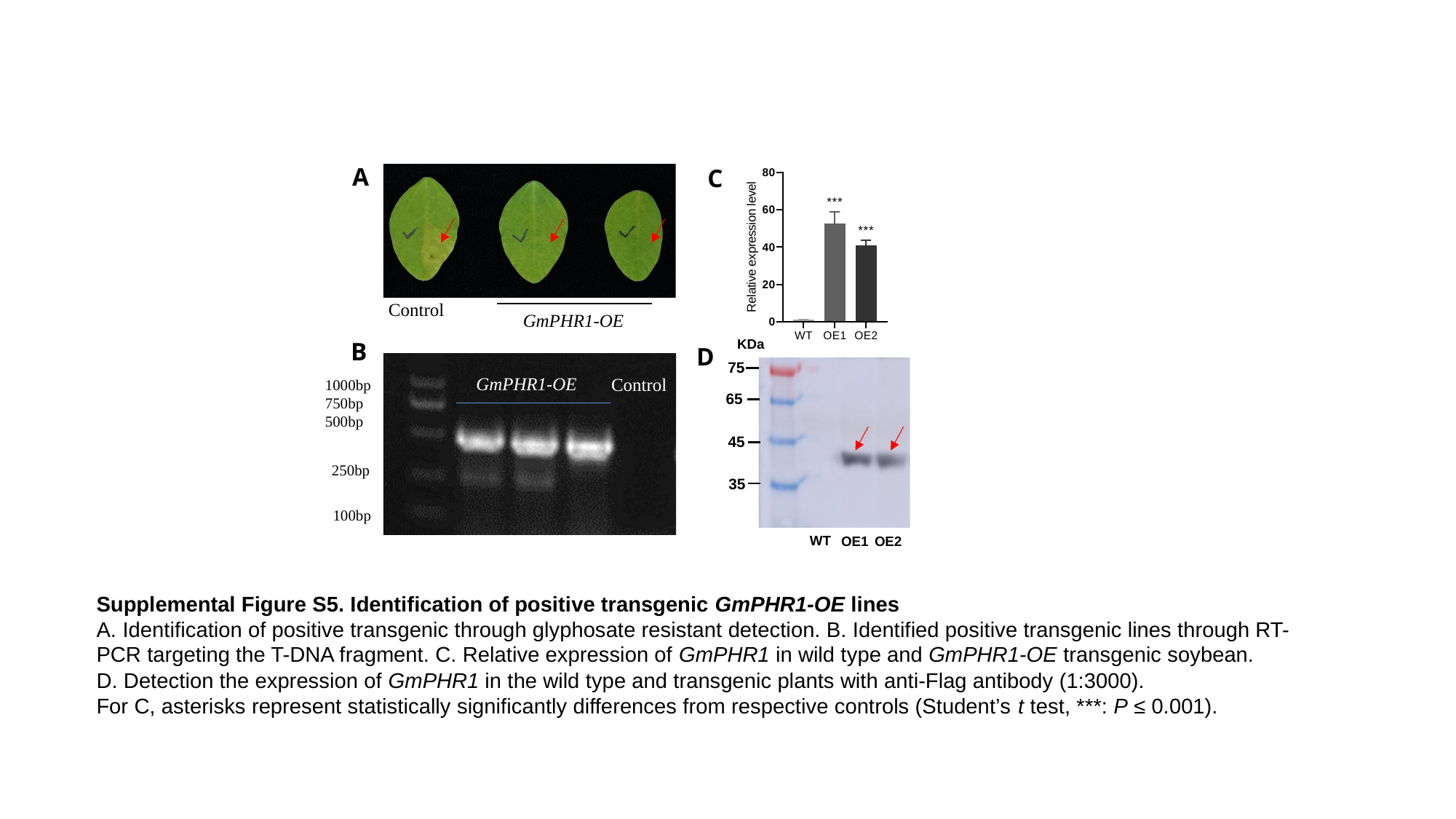

A
C
Control
GmPHR1-OE
KDa
75
65
45
35
WT
OE2
OE1
B
D
GmPHR1-OE
Control
1000bp
750bp
500bp
250bp
100bp
Supplemental Figure S5. Identification of positive transgenic GmPHR1-OE lines
A. Identification of positive transgenic through glyphosate resistant detection. B. Identified positive transgenic lines through RT-PCR targeting the T-DNA fragment. C. Relative expression of GmPHR1 in wild type and GmPHR1-OE transgenic soybean.
D. Detection the expression of GmPHR1 in the wild type and transgenic plants with anti-Flag antibody (1:3000).
For C, asterisks represent statistically significantly differences from respective controls (Student’s t test, ***: P ≤ 0.001).

## Slide 6
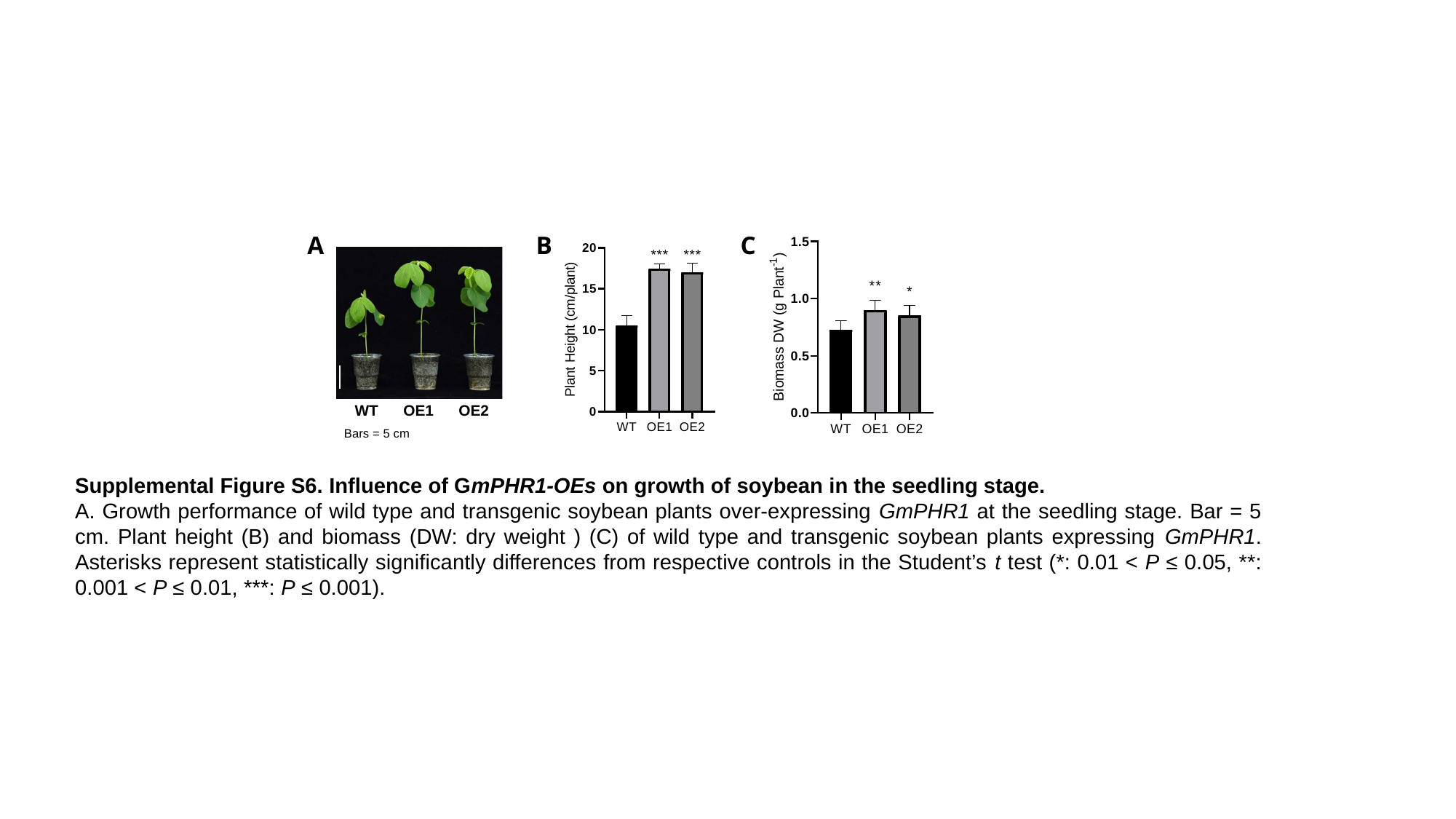

A
B
C
 WT OE1 OE2
Bars = 5 cm
Supplemental Figure S6. Influence of GmPHR1-OEs on growth of soybean in the seedling stage.
A. Growth performance of wild type and transgenic soybean plants over-expressing GmPHR1 at the seedling stage. Bar = 5 cm. Plant height (B) and biomass (DW: dry weight ) (C) of wild type and transgenic soybean plants expressing GmPHR1. Asterisks represent statistically significantly differences from respective controls in the Student’s t test (*: 0.01 < P ≤ 0.05, **: 0.001 < P ≤ 0.01, ***: P ≤ 0.001).

## Slide 7
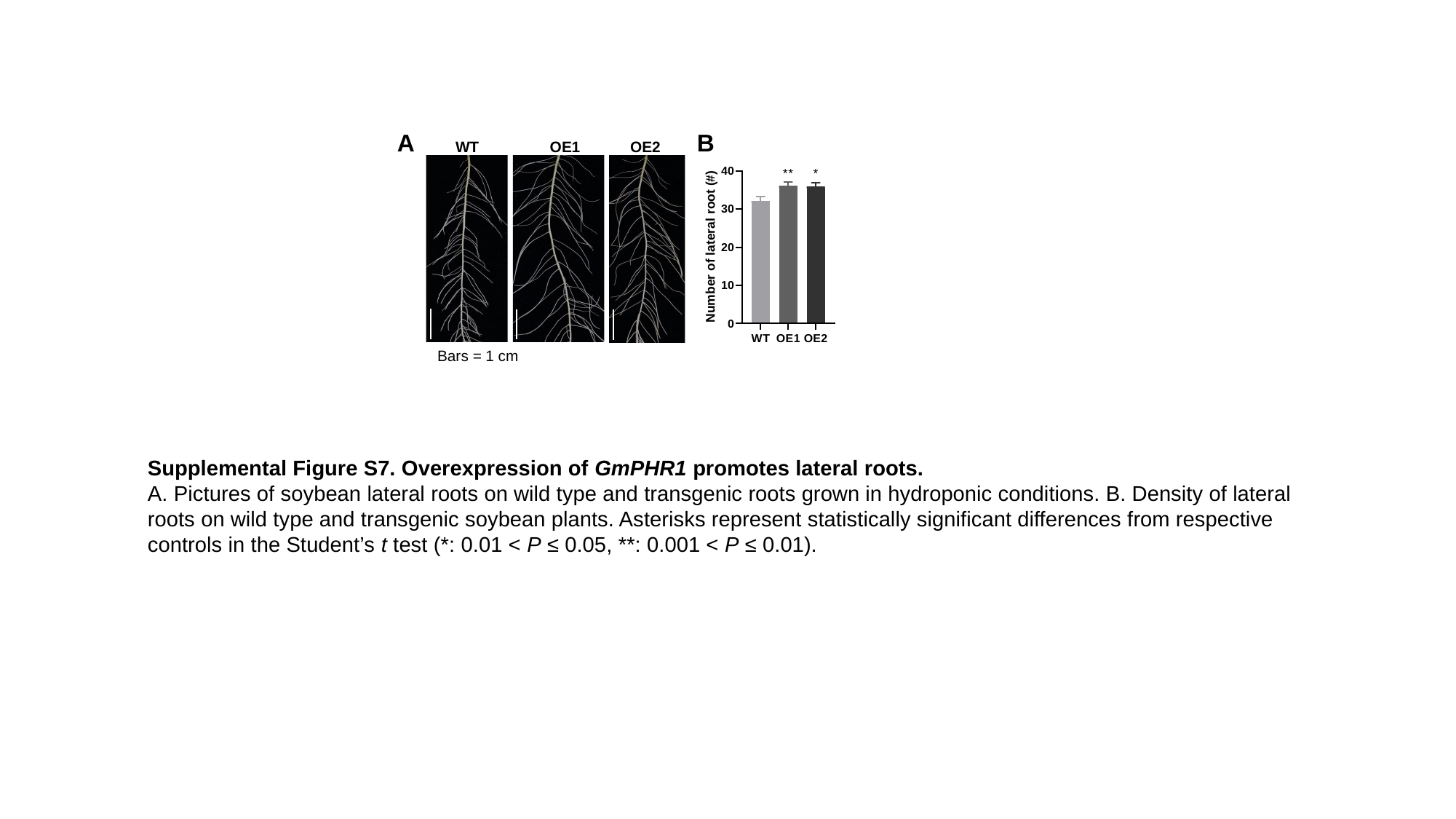

A
B
 WT OE1 OE2
Bars = 1 cm
Supplemental Figure S7. Overexpression of GmPHR1 promotes lateral roots.
A. Pictures of soybean lateral roots on wild type and transgenic roots grown in hydroponic conditions. B. Density of lateral roots on wild type and transgenic soybean plants. Asterisks represent statistically significant differences from respective controls in the Student’s t test (*: 0.01 < P ≤ 0.05, **: 0.001 < P ≤ 0.01).

## Slide 8
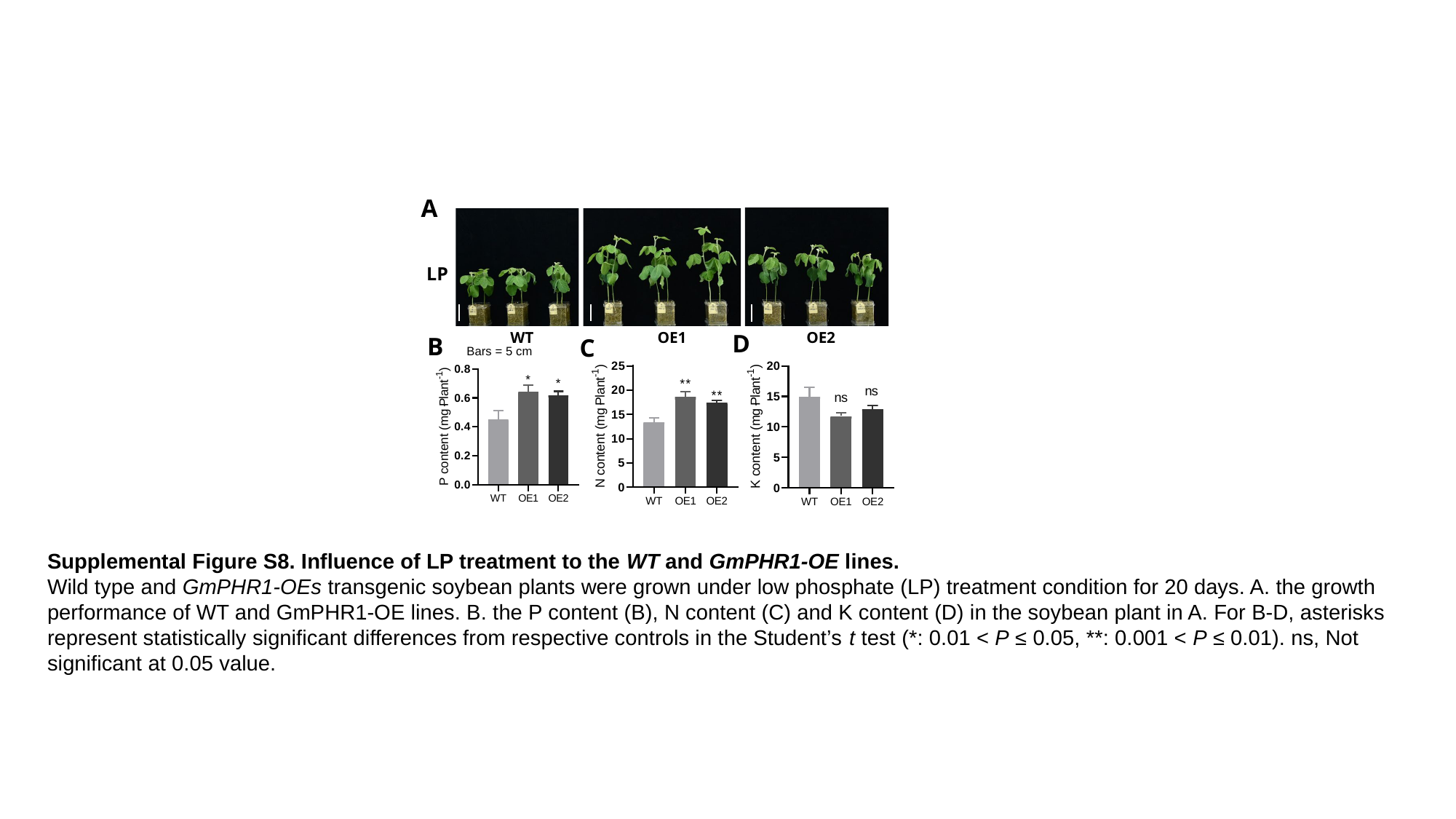

A
LP
 WT OE1 OE2
D
B
C
Bars = 5 cm
Supplemental Figure S8. Influence of LP treatment to the WT and GmPHR1-OE lines.
Wild type and GmPHR1-OEs transgenic soybean plants were grown under low phosphate (LP) treatment condition for 20 days. A. the growth performance of WT and GmPHR1-OE lines. B. the P content (B), N content (C) and K content (D) in the soybean plant in A. For B-D, asterisks represent statistically significant differences from respective controls in the Student’s t test (*: 0.01 < P ≤ 0.05, **: 0.001 < P ≤ 0.01). ns, Not significant at 0.05 value.

## Slide 9
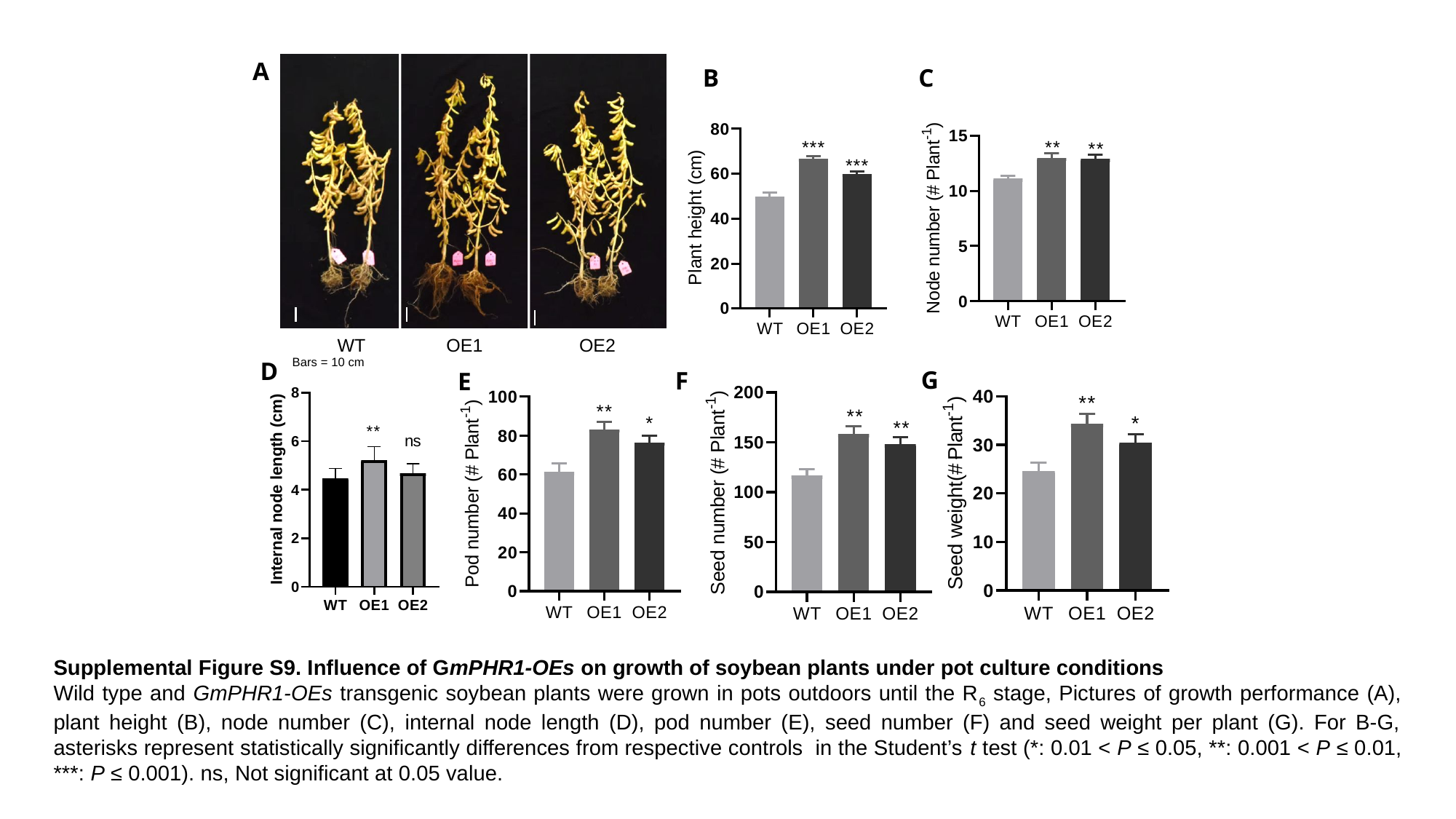

A
WT OE1 OE2
B
C
D
F
E
Bars = 10 cm
G
Supplemental Figure S9. Influence of GmPHR1-OEs on growth of soybean plants under pot culture conditions
Wild type and GmPHR1-OEs transgenic soybean plants were grown in pots outdoors until the R6 stage, Pictures of growth performance (A), plant height (B), node number (C), internal node length (D), pod number (E), seed number (F) and seed weight per plant (G). For B-G, asterisks represent statistically significantly differences from respective controls in the Student’s t test (*: 0.01 < P ≤ 0.05, **: 0.001 < P ≤ 0.01, ***: P ≤ 0.001). ns, Not significant at 0.05 value.

## Slide 10
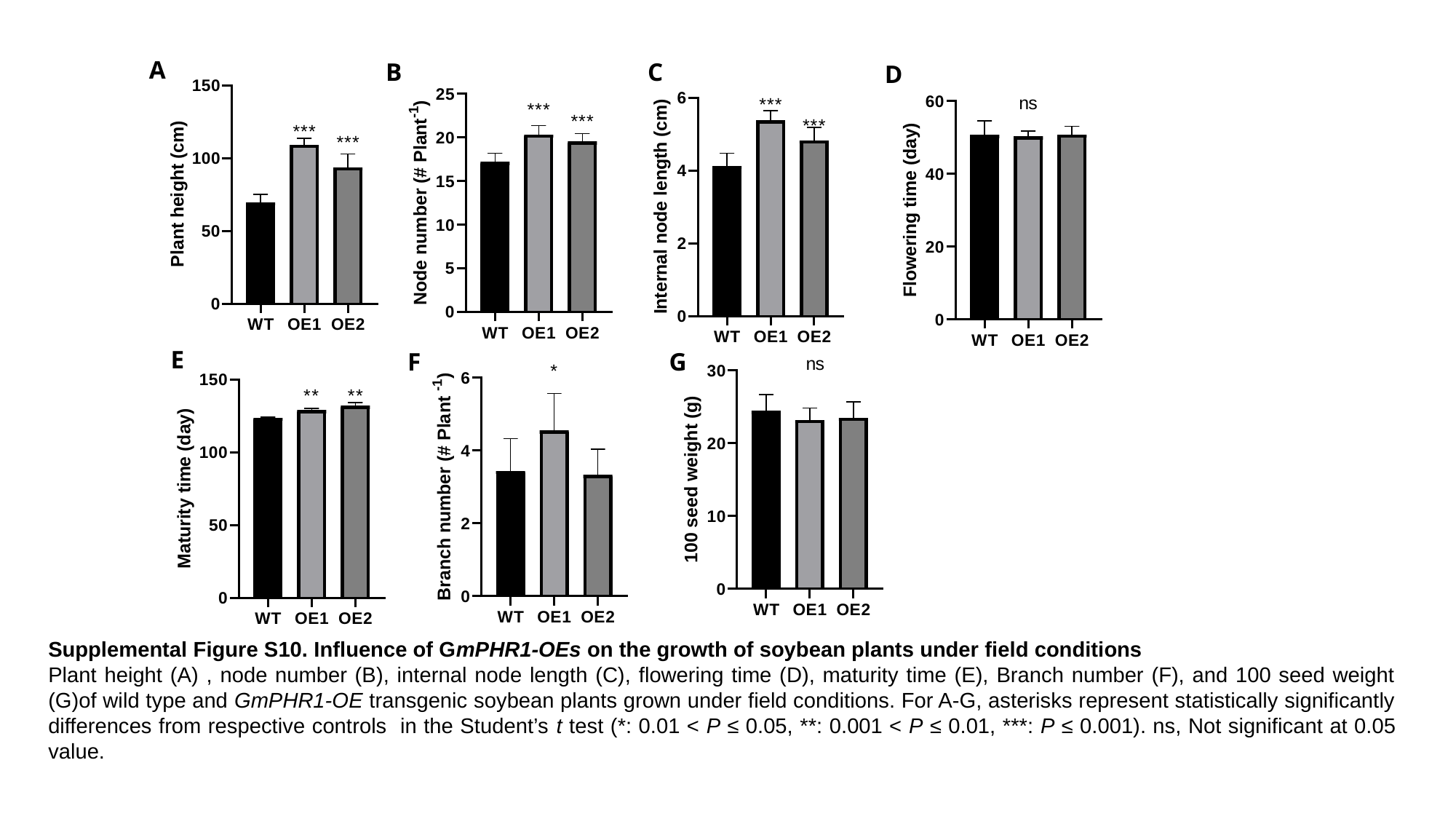

A
B
C
D
E
F
G
Supplemental Figure S10. Influence of GmPHR1-OEs on the growth of soybean plants under field conditions
Plant height (A) , node number (B), internal node length (C), flowering time (D), maturity time (E), Branch number (F), and 100 seed weight (G)of wild type and GmPHR1-OE transgenic soybean plants grown under field conditions. For A-G, asterisks represent statistically significantly differences from respective controls in the Student’s t test (*: 0.01 < P ≤ 0.05, **: 0.001 < P ≤ 0.01, ***: P ≤ 0.001). ns, Not significant at 0.05 value.
