# Supplementary material for: Overexpression of GmPHR1 Promotes Soybean Yield through Global Regulation of Nutrient Acquisition and Root Development
Source: Int J Mol Sci. 2022 Dec 3;23(23):15274. doi: 10.3390/ijms232315274 (PMC9740814; doi:10.3390/ijms232315274)
Supplement: Supplementary file 1 [file ijms-23-15274-s001.zip › Supplementary Table S1.pdf]

Supplementary Table S1 primers used for the vector construction and identification

Primers name	Primer sequence (5’-3’)
GmPHR1-qRT-F	AGGAAGTGCACAAAGAGAATAGT
GmPHR1-qRT-R	CCACCTGCAGATACAAGGTCATT
*GmPHR1*pro-F	GTAAGACGGACCCAAAGAACA
*GmPHR1*pro-R	TCACTCACTTCACACACTACAC
*GmPHR1*pro-GUS-F	GATTTGAAAAATCTCAGAATTCGTAAGACGGACCCAAAGAAC
*GmPHR1*pro-GUS-R	CCTCAGATCTACCATGGCGCGCCTCACTCACTTCACACACTAC
35S-F	ATGACGCACAATCCCACTATCC
GmPHR1-test-R	CAGAACACAACATCATTGGCT
GUS-test-R	TTTCTACAGGACGGACGAGT
GmPHR1-Blunt-F	GTGTAGTGTGTGAAGTGAGTGA
GmPHR1-Blunt-R	TCATTATTCAATCCAATGGTC
Bar-F	CACTATCCTTCGCAAGACC
Bar-R	CTAGGCTTAGTCTGCGGTGC
